# Management of Conduit Stenosis and Iatrogenic Aortopulmonary Window Post-Ross Procedure

**DOI:** 10.1055/a-2779-0604

**Published:** 2026-01-27

**Authors:** Stelios Ioannou, George Shiakos, Aphrodite Tzifa, Ioannis Tzanavaros

**Affiliations:** 1 Department of Pediatric and Adult Cardiac Surgery, Cardiac Innovation Center at Apollonion Private Hospital, Nicosia, Cyprus; 2 Medical School, University of Nicosia, Nicosia, Cyprus; 3 Medical School, European University, Nicosia, Cyprus; 4 Department of Congenital Heart Disease, Mitera Children's Hospital, Athens, Greece

**Keywords:** endocarditis, pulmonary valve, pulmonary arteries/veins, Ross procedure, homograft, xenograft, aortopulmonary window

## Abstract

This case report describes a 23-year-old male with a history of bicuspid aortic valve endocarditis managed with an emergency Ross procedure in 2020, whereby a xenograft pulmonary conduit prosthesis was implanted in the pulmonary position, followed by pulmonary conduit stenosis treated initially with high-pressure balloon dilatation 3 years later. The consequent diagnosis of iatrogenic aortopulmonary (AP) window, combined with severe recurrent pulmonary conduit stenosis, necessitated reoperation with right ventricle-to-pulmonary artery homograft replacement and repair of the AP shunt.

## Introduction

Ross procedure is a complex surgical technique involving autotransplantation of the patient's pulmonary valve into the aortic position. However, the procedure is not without long-term complications, among which is stenosis of the right ventricle–pulmonary artery (RVPA) conduit, which frequently necessitates repeated interventions. 1 2 Recurrent conduit stenosis can impose significant hemodynamic strain on the right ventricle, often requiring surgical revision or balloon angioplasty. The latter may be either ineffective or may develop iatrogenic vascular windows. Such shunts, though infrequently reported, may have profound clinical implications, as they create an abnormal circulatory pathway that exacerbates pressure and volume overload. 3

We report the case of a Ross patient with recurrent pulmonary conduit stenosis, who was diagnosed with an iatrogenic aortopulmonary (AP) connection after high-pressure balloon dilation of the pulmonary conduit. 4

## Case Presentation

This case report presents the medical history of a 23-year-old male who was diagnosed with a bicuspid aortic valve in 2020. Soon after, he underwent tonsillectomy, and he subsequently developed fever, leading to a diagnosis of aortic valve endocarditis according to Duke criteria. Blood cultures revealed *Kocuria rosea* . This diagnosis led to emergency surgery, whereby a Ross procedure was performed. A 27-mm right ventricle to pulmonary artery xenograft conduit (BioPulmonic Conduit™, Sygan Medical) was implanted in the pulmonary position. Although initially stable, xenograft conduit stenosis recurred within 2 years, and this was at the level of the distal conduit to pulmonary artery bifurcation anastomosis, prompting high-pressure balloon dilatation in February 2023, as per multidisciplinary decision.

**Fig. 1:**
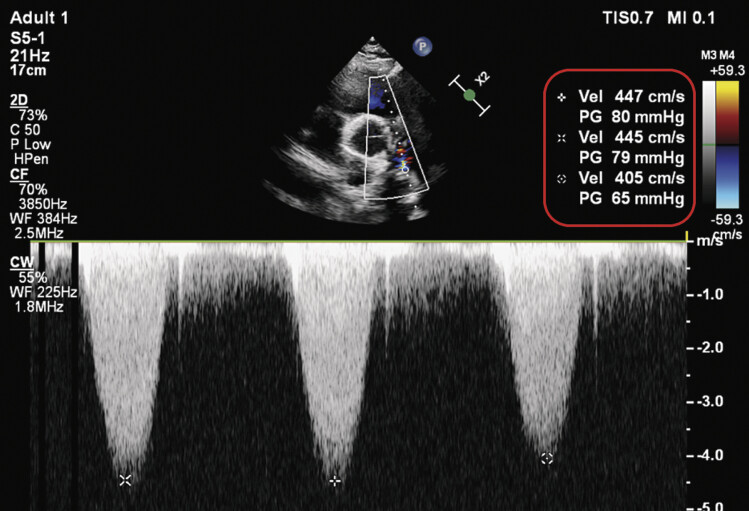
Preoperative echocardiography.

Despite temporary relief, the patient's symptoms of tachycardia and ventricular ectopy re-emerged until June 2024, along with a prominent diastolic murmur. Then an echocardiography confirmed restenosis of the pulmonary conduit with a peak gradient of 80 mm Hg ( Fig. 1 ), signs of right ventricular pressure overload, as evidenced by a dilated right ventricle as well as the D-shape morphology of the intraventricular septum. In addition, abnormal flow into the main pulmonary artery was also noted, which seemed to originate from the ascending aorta. In the absence of aortic regurgitation, holodiastolic flow reversal in the aortic arch supported the aforementioned finding. It was hypothesized that the AP shunt was likely of iatrogenic etiology caused during the high-pressure balloon dilatation performed a year earlier, whereas an endocarditis was ruled out. These findings prompted further investigation with magnetic resonance imaging (MRI).

**Fig. 2:**
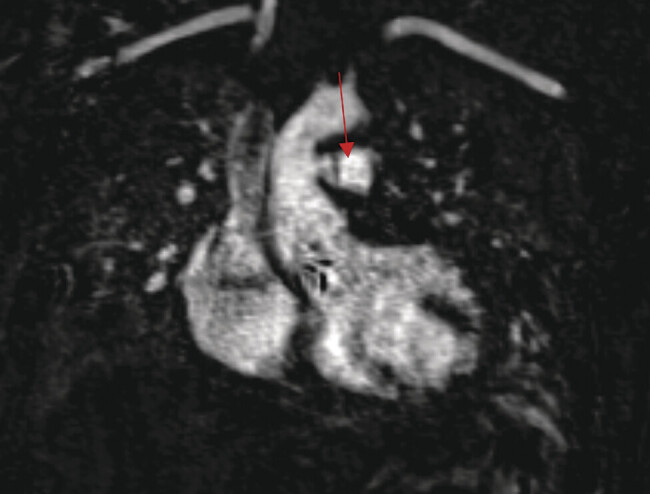
Cardiac MRI revealing the aortopulmonary connection. MRI, magnetic resonance imaging.

The MRI confirmed the presence of two sites of iatrogenic AP communications, one being between the ascending aorta and the main pulmonary artery, and the second one between the ascending aorta and the right pulmonary artery ( Fig. 2 ). Pulmonary-to-systemic blood flow ratio (Qp:Qs) measured 2.4:1, confirming the hemodynamic significance of the communications.

Covered right ventricular outflow tract stent followed by transcatheter pulmonary valve implantation was considered, but given the presence of two shunt sites, not amenable to simultaneous transcatheter closure, the multidisciplinary team recommended surgical intervention, including RVPA conduit replacement using a decellularized pulmonary valve homograft conduit and AP windows closure.

**Fig. 3:**
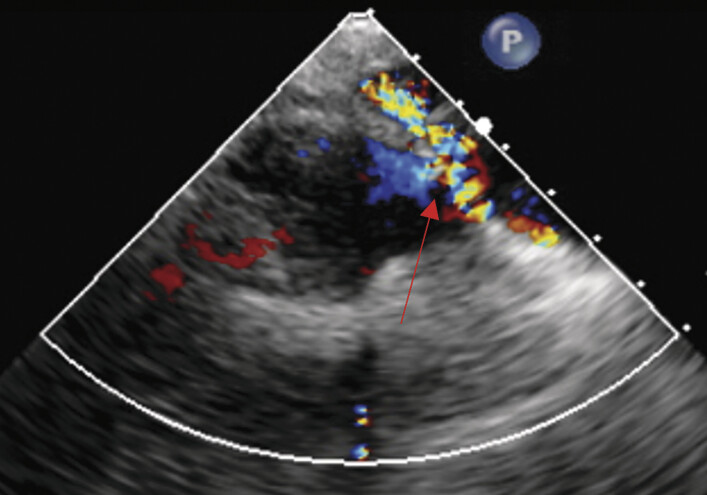
Intraoperative transesophageal echocardiography showing the aortopulmonary shunt.

**Fig. 4:**
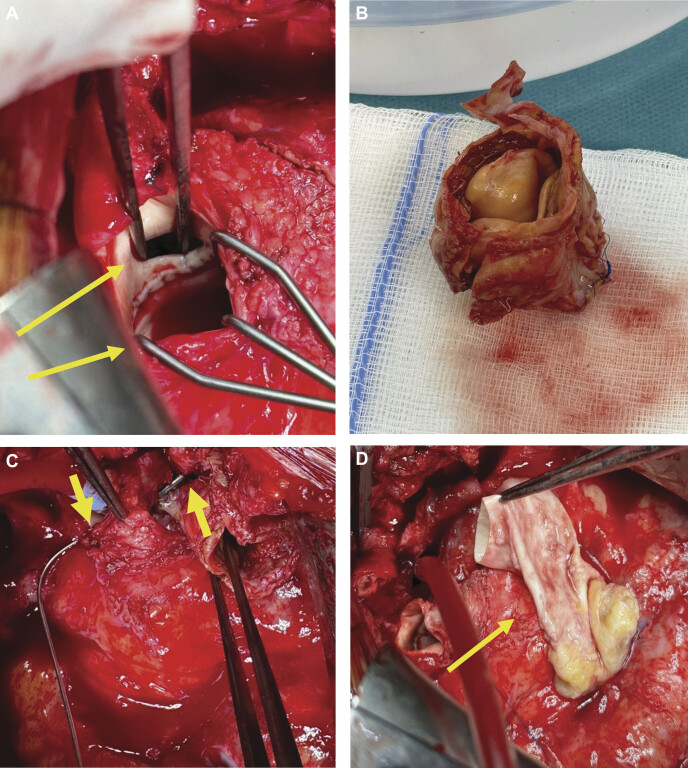
( **A** ) Upper arrow shows the native perforation of the aorta, and lower arrow shows the autograft. ( **B** ) The excised homograft conduit with apparent areas of calcification, in the diagnostic context of pulmonary stenosis. ( **C** ) Overhead view of the substantial aortopulmonary connection, as can be seen via the insertion of a metallic probe (see the arrows). ( **D** ) Decellularized pulmonary homograft conduit.

Intraoperative transesophageal echocardiography (TEE) confirmed the stenosis across the existing conduit and identified and confirmed the AP shunt ( Fig. 3 ). Cardiopulmonary bypass was initiated via cannulation of the right femoral vessels. Median resternotomy was performed to access the thoracic cavity. The stenotic pulmonary conduit was excised and replaced with a cryopreserved, decellularized homograft for RVPA reconstruction, and the fistula to the right pulmonary artery was directly closed. The perforated aorta–autograft AP connection was also directly closed ( Fig. 4A–D ). Intraoperative TEE following the repair confirmed resolution of the abnormal flow patterns.

The postoperative course was uneventful, and the patient was swiftly mobilized and discharged on the fifth postoperative day.

**Fig. 5:**
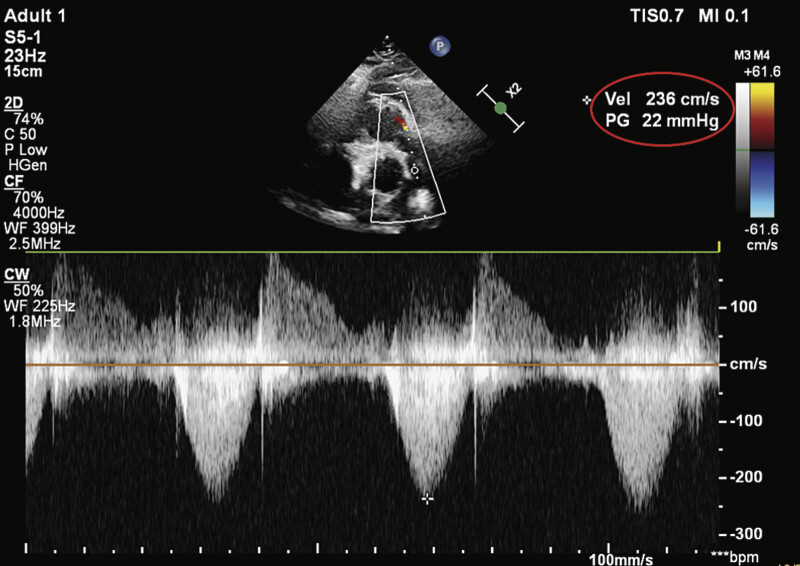
Postoperative echocardiography 30-day postoperative reveals the gradient across the pulmonary homograft.

Follow-up appointments were scheduled on the 7th day postdischarge as well as on the 30th day postsurgery. No complications were mentioned within this time interval, where the echocardiography 30-day postsurgery demonstrated resolution of the aforementioned stenosis ( Fig. 5 ).

## Discussion

Recurrent conduit stenosis is one of the most frequently encountered complications following the Ross procedure, often arising from a combination of factors, including graft material degradation and neointimal proliferation. Decellularized grafts, frequently employed for pulmonary conduits, have shown a propensity for intimal hyperplasia and calcification, contributing to conduit failure and the need for reintervention. Various studies highlight that up to 50% of patients may experience conduit dysfunction within 10 to 15 years postoperatively, underlining the long-term maintenance challenges associated with the Ross procedure. 5

While transcatheter balloon angioplasty offers a minimally invasive approach for stenotic conduit relief, it poses risks such as iatrogenic injury with an ultimate necessity for surgical repair to restore hemodynamic stability and reduce ventricular strain. To our knowledge, this is the first report on an AP window after a successful Ross operation. 6

The role of interdisciplinary planning cannot be overstated in complex post-Ross patients. Surgeons, cardiologists, and radiologists must collaborate closely to balance the risks of interventional versus surgical strategies.

## Conclusion

This case of iatrogenic AP shunts in a post-Ross patient underscores the complexities associated with managing concurrent pulmonary conduit restenosis in this cohort. Although transcatheter interventions may provide temporary relief from stenosis, they carry risks that can exacerbate vascular anomalies, leading to the ultimate defect resolution through a surgical procedure.
